# Light Emission from M-Type Enantiomer of 2,13-bis(hydroxymethyl)[7]-thiaheterohelicene Molecules Adsorbed on Au(111) and C_60_/Au(111) Surfaces Investigated by STM-LE

**DOI:** 10.3390/ijms232315399

**Published:** 2022-12-06

**Authors:** Paweł Krukowski, Takuma Hattori, Megumi Akai-Kasaya, Akira Saito, Hideji Osuga, Yuji Kuwahara

**Affiliations:** 1 Department of Solid State Physics, Faculty of Physics and Applied Informatics, University of Lodz, 90–236 Łódź, Poland; 2 Department of Precision Engineering, Graduate School of Engineering, Osaka University, Suita 565–0871, Japan; 3 Department of Materials Science and Chemistry, Faculty of Systems Engineering, Wakayama University, Wakayama 640-8510, Japan

**Keywords:** helicene, STM-LE, Au(111), buffer layer

## Abstract

Light emission from the M-type enantiomer of a helicene derivative (2,13-bis(hydroxymethyl)[7]-thiaheterohelicene) adsorbed on the clean Au(111) and the C_60_-covered Au(111) surfaces were investigated by tunneling-current-induced light-emission technique. Plasmon-originated light emission was observed on the helicence/Au(111) surface and it was strongly suppressed on the area where the helicene molecules were adsorbed at the edges of the Au(111) terraces. To avoid luminescence quenching of excited helicene molecules and to suppress strong plasmon light emission from the Au(111) surface, C_60_ layers were used as decoupling buffer layers between helicene molecules and the Au(111) surface. Helicene molecules were adsorbed preferentially on the Au(111) surface rather than on the C_60_ buffer layers due to the small interaction of the molecules and C_60_ islands. This fact motivated us to deposit a multilayer of helicene molecules onto the C_60_ layers grown on the Au(111) surface, leading to the fact that the helicene/C_60_ multilayer showed strong luminescence with the molecules character. We consider that such strong light emission from the multilayer of helicene molecules has a plasmon origin strongly modulated by the molecular electronic states of (M)-[7]TH-diol molecules.

## 1. Introduction

In recent years, considerable effort has been devoted to investigating molecular light emission at nanoscale by scanning-tunneling-microscope-based light emission technique (STM-LE) due to its high spatial resolution [1,2,3,4,5,6,7,8,9,10,11,12,13,14,15,16,17,18]. For instance, the vibronically resolved intramolecular photon emission from a single magnesium porphine (MgP) molecule adsorbed on Al_2_O_3_ grown on NiAl(110) was detected [12]. However, despite many STM-LE investigations on different molecular species, the actual emission mechanism is still controversial [14]. This is because the localized surface plasmons (LSP) are excited by inelastic tunneling electrons at the nanocavity between STM tip and metal surface, followed by radiative decay with high quantum efficiency. Distinguishing weak molecular luminescence from superimposed strong plasmon-mediated light emission is not a trivial task. Moreover, luminescence from molecules adsorbed directly on a metal substrate is strongly reduced due to the energy transfer from the excited molecular state to the metal substrate (radiation-less de-excitation of the molecules). To avoid such energy quenching and to suppress strong plasmon light emission from a bare metal surface, some electronically decoupling buffer layers such as Al_2_O_3_ [1,2,11], C_60_ [3], graphene [6], NaCl [2,7,8,9,10], and molecular multilayers [11] were proposed.

The investigation of various helicene molecules has attracted significant attention owing to their unusual chiroptical properties and a wide prospect of application in many fields including optoelectronic devices such as organic light-emitting diode and organic field-effect transistor [19,20,21,22,23]. In particular, interest has been aroused in the imaging of stereochemical recognition of carbohelicene molecules on metal surfaces at molecular scale using STM to better understand the fundamental rules responsible for racemate and conglomerate crystallization [24]. Since we are interested in the detection of circularly polarized luminescence (CPL) from chiral molecules, thus far, we have investigated thiaheterohelicene molecules and their derivatives using STM; Chaunchaiyakul et al. investigated adsorption of M-type enantiomer of 2,13-bis(hydroxymethyl)[7]-thiaheterohelicene molecules on Au(111) showing formation of self-assembled zigzagged twin rows [25]. Chaunchaiyakul et al. reported a chemical reaction occurring during tip-enhanced Raman spectroscopy measurements on racemic mixture of 2,13-bis(aldehyde)[7]thiaheterohelicenes (rac-[7]TH-dial) molecules adsorbed on Au(111) [26]. Krukowski et al. showed the results of STM-tip-induced light emission on molecules rac-[7]TH-dial molecules adsorbed on Au(111), Cu(001), and NiAl(110) surfaces, revealing the strong enhancement of light emission over rac-[7]TH-dial clusters formed on NiAl(110) [5]. In addition, Krukowski et al. have shown that crystallization of a racemic mixture of [5] and [7]thiaheterohelicene on the Ag(111) surface into highly ordered molecular domain was strongly influenced by stereospecific interactions [27]. The crystallization of a racemic mixture of [5] and [7]thiaheterohelicene molecules showing two-dimensional highly ordered molecular domains on a Ag(111) surface was investigated by STM at 80 K. The observed crystallization of both thiaheterohelicene molecules was strongly affected by stereospecific interactions resulting from different numbers of aromatic rings incorporated into the helical skeletons of molecules.

**Figure 1:**
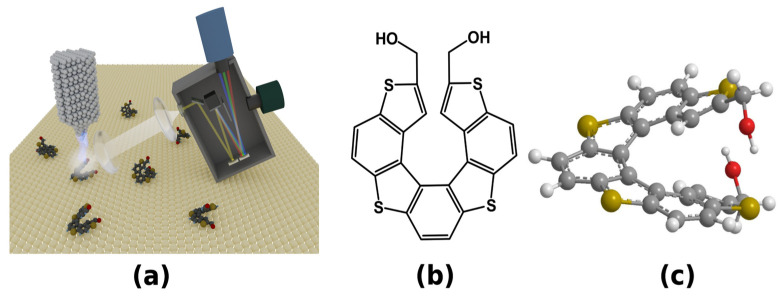
(**a**) Schematic illustration of STM-LE equipment. Local light emission induced in tunneling junction on molecule/substrate system is collected through lens and transmitted to photodetectors. (**b**) Molecular structure of 2,13-bis(hydroxymethyl)[7]-thiaheterohelicene and (**c**) its three-dimensional view of M-type enantiomer ((M)-[7]TH-diol). The red, gray, white, and yellow balls indicate O, C, H, and S atoms, respectively.

In this study, we report the results of STM-LE investigations of the M-type enantiomer of a helicene derivative, namely, 2,13-bis(hydroxymethyl)[7]-thiaheterohelicene (hereafter as (M)-[7]TH-diol) deposited on Au(111) and C_60_ grown on Au(111). Note that the Au(111) is a model surface for adsorption of various molecules in STM study [28,29,30]. Figure 1a shows the schematic illustration of STM-LE equipment. Local light emission over molecule/surface system induced by an STM tip can be collected through lens and then transmitted to photodetectors such as CCD camera and photomultiplier.

The strong light emission from the (M)-[7]TH-diol multilayer adsorbed on C_60_ layers grown on the Au(111) surface was observed, whose emission spectra contained the fine structures with a molecular emission character. We consider that the strong light emission from the (M)-[7]TH-diol multilayer on C_60_/Au(111) surface is plasmon-originated and it was strongly affected by vibronic transitions in (M)-[7]TH-diol molecules. Figure 1b shows the molecular structure of the [7]TH-diol molecule, while Figure 1c shows the three-dimensional view of (M)-[7]TH-diol stabilized in the gas phase and calculated by density functional theory (DFT). The geometry of (M)-[7]TH-diol molecule were obtained from DFT simulation using the standard B3LYP hybrid density functional combined with 6–311++G(d,p) basis sets implemented in the Gaussian09 program. 

## 2. Results and Discussion

### 2.1. STM-LE Investigation of (M)-[7]TH-diol Molecules Adsorbed on Au(111)

**Figure 2:**
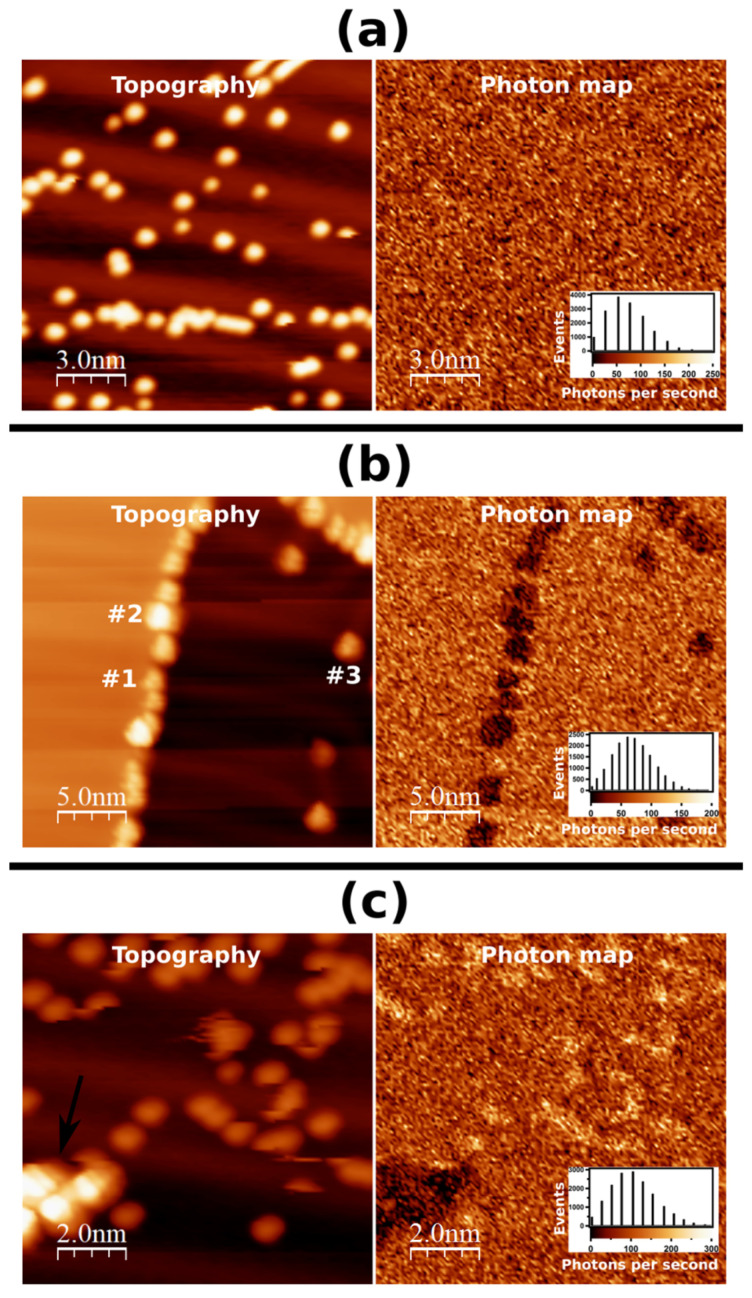
(**a**) STM topographic image and simultaneously acquired photon integration map of isolated (M)-[7]TH-diol molecules adsorbed on the Au(111) surface (−3.5 V, 500 pA, 128 × 128 pixels, 39 ms/pixel). (right column) The photon emission per second taken at each pixel of the photon integration map was used to obtain the histogram. The histogram shows a weak emission of about 50 photons per second. (**b**) STM topographic image and simultaneously acquired photon integration map of clusters of (M)-[7]TH-diol molecules preferentially adsorbed at the step edges of Au(111) terraces (−3.0 V, 250 pA, 128 × 128 pixels, 7.8 ms/pixel). (right column) The histogram shows a weak emission of about 60 photons per second. (**c**) STM topographic image and simultaneously acquired photon integration map of isolated (M)-[7]TH-diol molecules adsorbed on Au(111) surface (−3.0 V, 500 pA, 128 × 128 pixels, 39 ms/pixel), in which meaningful enhancement of light emission on the clusters are observed. (right column) The histogram shows emission of 100 photons per second.

Figure 2 shows high-resolution STM topographic images of isolated (M)-[7]TH-diol molecules and clusters adsorbed on the clean Au(111) surface and corresponding photon integration maps. The details related to the adsorption and self-assembly formation from (M)-[7]TH-diol molecules on the clean Au(111) surface were found in our previous paper [25]. At a low coverage the (M)-[7]TH-diol molecules were preferentially adsorbed at the step edges of the terraces and at the elbows of the herringbone reconstruction on Au(111). With increasing the coverage of (M)-[7]TH-diol molecules, the formation of highly ordered islands was observed. The image of Figure 2a shows that despite the relatively high tunneling current (500 pA) and high bias voltage (−3.5 V) set point, single (M)-[7]TH-diol molecules were clearly imaged. The mean diameter and mean apparent heights of the bright protrusion ascribed to single molecules were estimated to be 0.9 ± 0.1 and 0.12 ± 0.02 nm, respectively. The mean diameter is in good agreement with the size of single (M)-[7]TH-diol molecule in the STM image. The photon integration map presented in Figure 2a (right) was acquired simultaneously with the topography image of Figure 2a (left). The photon integration map shows homogeneous light emission and lack of any correlation with the topography image. The light emission was caused by the radiative decay of the plasmon between the tip and Au(111) (tip induced plasmon: TIP) through inelastic tunneling processes. The light emission was not suppressed or enhanced by the presence of isolated (M)-[7]TH-diol molecules adsorbed on the Au(111) surface. Note that the suppression of light emission on molecule should be visible as a darker area on the photon integration map in comparison to bare Au(111) surface. On the other hand, the enhancement of light emission on molecule should be visible as a brighter area on the photon integration map. From the histogram of photon map image presented in Figure 2a (right column), the light emission was small at the bias voltage of −3.5 eV. The STM topographic image of Figure 2b (left) shows single molecules (marked like as #1) and clusters (marked like as #2) of (M)-[7]TH-diol preferentially adsorbed at the step edges of the Au(111) surface. The corresponding photon integration map shows excellent correlation with the topographic image at the bias voltage of −3.5 eV, indicating the suppression of light emission on the molecules, i.e., the light intensity was approximately 3 times smaller on the area covered with (M)-[7]TH-diol molecules at the step edges than that on flat Au(111) surface. The light emission on the cluster (marked like as #3) adsorbed on the flat part of Au(111) was also strongly suppressed. 

It should be emphasized that we occasionally observed light emission enhancement on Au(111) covered with (M)-[7]TH-diol molecules, as seen in Figure 2c. The light intensity on the area covered with single (M)-[7]TH-diol molecules was approximately 2 times larger than that on the Au(111) surface. However, such an enhancement of the light emission was rarely observed suggesting the change of a tip apex condition. Note that the plasmon light emission intensity from an aggregation of clusters (marked by arrow) was strongly suppressed.

### 2.2. C_60_ Buffer Layer Grown on Au(111)

**Figure 3:**
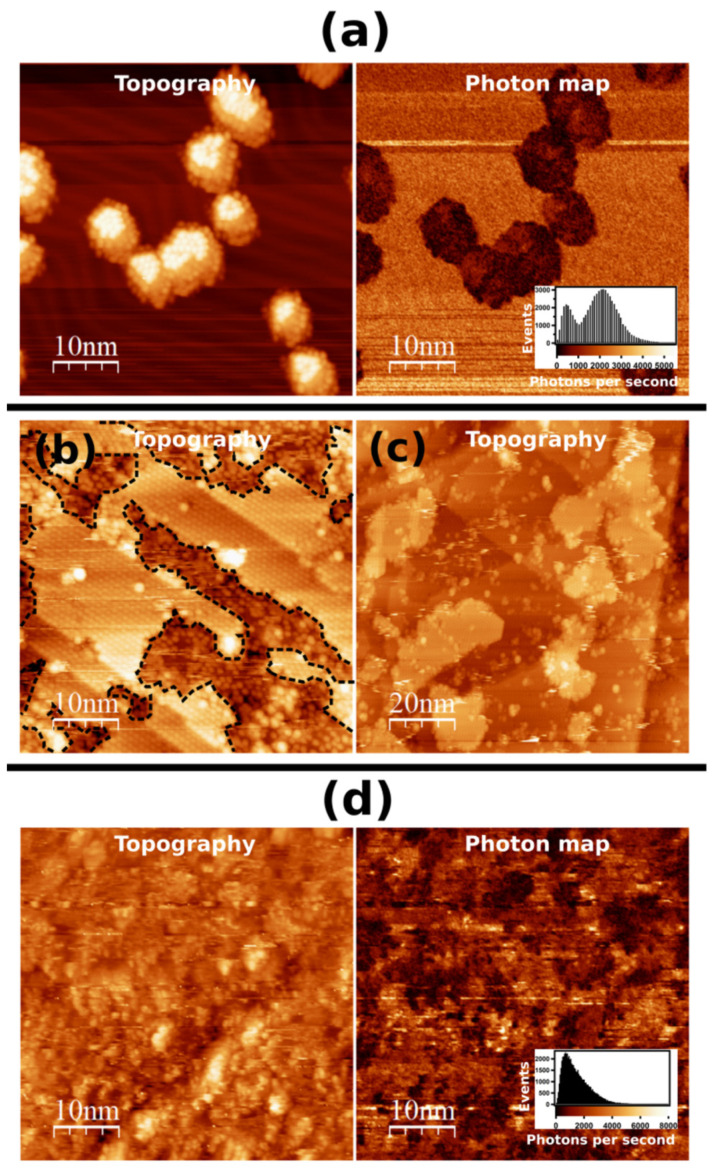
(**a**) STM topographic image and simultaneously acquired photon integration map of C_60_ islands grown on Au(111) surface (−3.5 V, 500 pA, 256 × 256 pixels, 9.8 ms/pixel). (**b**) STM topographic image of (M)-[7]TH-diol molecules deposited on Au(111) surface partially covered with C_60_ islands (1.0 V, 50 pA). C_60_ islands are surrounded by the dotted lines. (**c**) STM topography image of (M)-[7]TH-diol molecules at a low coverage deposited on fully covered C_60_ layer grown on Au(111) surface (−2.5 V, 50 pA). (**d**) STM image (−3.0 V, 800 pA, 256 × 256 pixels, 15.6 ms/pixel) and simultaneously acquired photon integration map of multilayer of (M)-[7]TH-diol adsorbed on fully covered C_60_ layer grown on Au(111) surface.

Figure 3a shows the STM topographic image after the deposition of C_60_ at a low coverage. Owing to the low temperature of the surface during the C_60_ deposition, the formation of C_60_ islands in the flat Au(111) terraces was observed. The STM topographic image (Figure 3a left) shows the growth of hexagonally ordered C_60_ islands with single and double monolayer heights. The mean apparent heights of the 1st and 2nd layers were estimated to be 0.5 ± 0.1 and 0.9 ± 0.1 nm, respectively. The photon integration map in Figure 3a (right) simultaneously acquired with the STM image clearly shows the contrast between the light emission on the Au(111) substrate and that on C_60_ islands. The light intensity observed on the 1st layer of the C_60_ islands was approximately 4 times smaller than that on the Au(111) surface. Figure 3b shows the STM topography image of the Au(111) surface partially covered with C_60_ islands following the deposition of (M)-[7]TH-diol molecules. It can be seen that the (M)-[7]TH-diol molecules preferentially adsorbed on the Au(111) surface rather than on the C_60_ islands. Figure 3c shows an STM topography image of the Au(111) surface fully covered with C_60_ layers after the deposition of (M)-[7]TH-diol molecules at a low coverage. Since adsorbates of isolated (M)-[7]TH-diol molecules and clusters on the C_60_ layers were observed, we carried out light emission investigation on such a sample. However, at the high tunneling current, which is necessary for light emission investigation, we could not obtain a high resolution STM image of the (M)-[7]TH-diol molecules and clusters by the modification of the surfaces. Therefore, we deposited a multilayer of (M)-[7]TH-diol molecules on C_60_ layers grown on the Au(111) surface presented in Figure 3c. The multilayer of (M)-[7]TH-diol molecules on C_60_ layers can be seen in image presented in Figure 3d (left). It is easy to see that the multilayer of (M)-[7]TH-diol molecules deposited on C_60_ shows a disordered character. The photon integration map presented in Figure 3d (right) indicates a high and inhomogeneous light emission. It was found that the correlation between the topography image and the photon integration map is rather weak. 

Keeping in mind that the plasmon light emission should be strongly suppressed owing to the increase in the tip-sample distance and the reduction in the electromagnetic coupling between the tip and the metal substrate resulting from the C_60_ and molecular layers [31], our obtained results seems to be extraordinary. This is because very strong light emission from the multilayer of (M)-[7]TH-diol molecules adsorbed on C_60_ layers grown on the Au(111) surface was obtained. The strong light emission suggests that C_60_ and molecular layers are effective enough to prevent the energy quenching of the excited molecules as for the molecular emission, or a new process responsible for the strong light emission occurs. 

**Figure 4:**
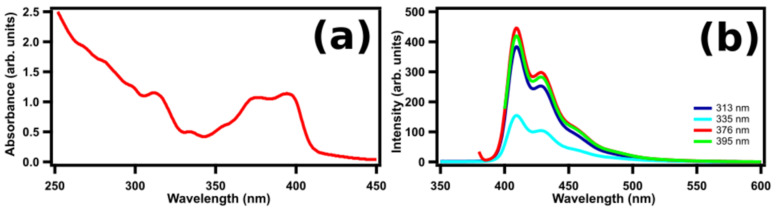
(**a**) UV-Vis absorption and (**b**) PL spectra of (M)-[7]TH-diol molecules in CHCl_3_ with concentration of 1.0 × 10^−5^ M. Both UV–Vis absorption and PL spectra indicate the Franck-Condon vibronic progression.

To identify the origin of the light emission, we compare STM-LE spectra with conventional laser-induced photoluminescence (PL) and ultraviolet–visible (UV–Vis) absorption spectra. Figure 4 shows (a) UV-Vis and (b) PL spectra of (M)-[7]TH-diol molecules dissolved in CHCl_3_ at a concentration of 1.0 × 10^−5^ M. The Franck–Condon vibronic progressions can be clearly observed both in UV–Vis and PL spectra and the mirror symmetry between absorption and PL spectra is seen as well. The absorption spectrum has an onset at 412 nm, two peaks at 396 and 375 nm, and a shoulder peak at 359 nm. HOMO/LUMO optical energy gap deduced from the absorption edge is estimated to be 3.0 eV. PL spectra show that the (M)-[7]TH-diol molecules dissolved in CHCl_3_ emit blue light with two emission peaks at 409 and 430 nm and a shoulder peak at 454 nm. 

**Figure 5:**
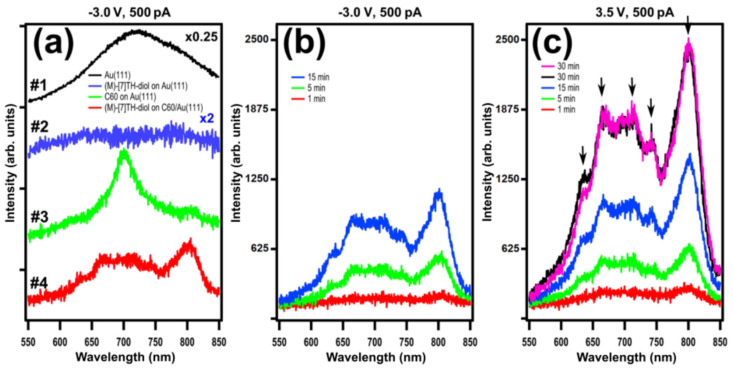
(**a**) STM-LE spectra taken on: Au(111) surface (#1), (M)-[7]TH-diol molecules adsorbed on Au(111) surface (#2), C_60_ layer grown on Au(111) (#3) surface and the multilayer of (M)-[7]TH-diol molecules adsorbed on C_60_ layer grown on Au(111) surface (#4). All spectra were obtained under the same condition at the sample bias of −3.0 V and tunneling current of 500 pA with acquisition time equal to 5 min. The spectra are vertically offset for visual clarity. (**b**) STM-LE spectra taken on the multilayer of (M)-[7]TH-diol molecules adsorbed on C_60_ layer grown on Au(111) surface. All spectra were obtained at a sample bias of −3.0 V and a tunneling current of 500 pA with different acquisition times. (**c**) STM-LE spectra taken on the multilayer of (M)-[7]TH-diol molecules adsorbed on C_60_ layer grown on Au(111) surface. All spectra were obtained at a positive sample bias of 3.5 V and a tunneling current of 500 pA with different acquisition times.

Figure 5a shows STM-LE spectra taken from the clean Au(111) surface (curve #1), (M)-[7]TH-diol molecules adsorbed on the Au(111) surface (curve #2), C_60_ island grown on Au(111) (curve #3), and (M)-[7]TH-diol molecules adsorbed on C_60_ island grown on Au(111) surface (curve #4). All spectra were obtained under the same conditions: at sample bias of −3.0 V and tunneling current of 500 pA with an acquisition time of 5 min during the STM raster scan on the area of 50 × 50 nm^2^. All spectra are raw ones with the appropriate amplification of the intensities for supporting the comparison among each other. On the clean Au(111) surface, we observed a broad spectrum centred at 720 nm, which is ascribed to a typical TIP light emission on the Au(111)-Pt/Ir tip junction [32]. Curve #2 shows reduced TIP light emission from the (M)-[7]TH-diol adsorbed on Au(111). Such suppression of plasmon light emission due to the presence of molecules (acting as a dielectric spacer) is interpreted by an increase in the tip-sample separation distance. Curve #3 shows the spectra from the C_60_ island adsorbed on Au(111). This result coincides well with the previous study in which the suppressed light emission from C_60_ grown on Au(111), indicating the pure plasmon emission modulated by the C_60_ clusters [33]. Note that one can see a peak at 700 nm corresponding to C_60_ molecular states involved in the inelastic tunneling process. This allows us to conclude that the layer of C_60_ can be used as a good decoupling buffer layer, leading to the reduction of the interaction between the deposited molecules and the metal surface of Au(111). Spectrum #4 shows light emission from the multilayer of (M)-[7]TH-diol adsorbed on C_60_ layers grown on the Au(111) surface. The spectrum shows a sharp emission peak at 800 nm with broad spectral features 600–750 nm region.

Figure 5b shows STM-LE spectra taken on the multilayer of (M)-[7]TH-diol adsorbed on C_60_/Au(111). All spectra were obtained under the same conditions: at a sample bias of −3.0 V and a tunneling current of 500 pA with different acquisition times ranging from 1 to 15 min with the STM raster scan on the area of 50 × 50 nm^2^. All spectra were really similar, both with the shape of envelop and any of peak positions among them. We also took STM-LE spectra from the multilayer of (M)-[7]TH-diol adsorbed on C_60_ grown on Au(111) at a positive sample bias of 3.5 V with different acquisition times ranging from 1 to 30 min (Figure 5c). We found that spectra obtained for positive bias voltage have well coincided with that for the negative bias voltage. Moreover, excellent reproducibility of spectra shape and peaks were found except for the integrated intensities depending on the acquisition time.

Since our STM-LE spectra taken on the multilayer of (M)-[7]TH-diol adsorbed on C_60_ grown on the Au(111) surface were apparently different from the PL spectra (Figure 4b), it is difficult to assign the STM-LE spectra to a pure molecular light emission. The obtained peaks of the PL spectra were attributed to the vibronic progression related to the S_1_(0–0) transition at 412 nm, obtained as the onset of the absorption spectra (S_1_ is the first singlet excited state of the molecule, the numbers in parentheses denote the vibronic levels in the initial and final states). The peaks at 410 nm (3.02 eV), 430 nm (2.88 eV), and 455 nm (2.74 eV) were assigned to the S_1_(0-*n*) (*n* = 0–2) transition. Note that the energy intervals correspond to the energy of stretch oscillation of benzene rings ~0.15 nm [17]. In the STM-LE spectra, some of the meaningful peaks of the molecules were obtained, for example, at 633, 665, 713, 741, and 803 nm (shown as the arrows in the spectra), whose corresponding photon energy was 1.96, 1.86, 1.74, 1.67, and 1.54 eV, respectively. No such complicated spectra was observed on the clean Au(111) and C_60_/Au(111) surfaces, such that the characteristic of the spectra in Figure 5b,c were considered to be strongly modified by the presence of (M)-[7]TH-diol molecules at the tunneling gap. We assumed that the broad peak at 800 nm might be assigned to the tail of pure TIP light emission and the other fine and sharp peaks are ascribed to the resonated plasmon peaks modulated by the molecular electronic states. It is difficult to identify the origin of the observed peaks in the (M)-[7]TH-diol molecules adsorbed on C_60_/Au(111) without any rules among the peaks such as the vibronic progression. The difference of the spectra between the rac-[7]TH-dial and the (M)-[7]TH-diol molecules might be caused by the different HOMO/LUMO gaps of the two molecules and also by the different resonation processes of the molecules with the electromagnetic field generated by TIP. 

There are a few main processes that may be responsible for the modification of plasmon light emission in the presence of a molecule: (a) Inelastic, off-resonance tunneling to the tail of broadened molecular states, elastic resonant tunneling and subsequent inelastic decay to the metal states, inelastic resonant tunneling of hot electrons, etc., [15], (b) excitation of dipole oscillation of adsorbed molecule by tunneling electrons and high electric field of STM junction leading to the enhancement of the local electromagnetic field allowing molecular excitation [34], and (c) molecular states of adsorbed molecules in a tunneling junction acting as spatially and energetically well-defined nanogates for plasmon excitations [3]. Consequently, the emission mechanism of STM-LE from molecules adsorbed on the metal surfaces strongly depends on the molecules, substrate surfaces, tip elements, and so on. Unfortunately, the details of the modification of plasmon light emission in (M)-[7]TH-diol molecules on C_60_/Au(111) are unclear from the data acquired only by the current experiment and require further investigations and comparisons with related molecular systems.

## 3. Materials and Methods

We used a low-temperature ultrahigh-vacuum STM system USM1400 (Unisoku Co., Hirakata, Japan) controlled by Nanonis BP 4.5 in combination with a high-voltage amplifier (RHK-SPM 100). Light emission induced by electrons tunneling through the STM tunneling junction was collected and parallelized by a plane-convex lens mounted inside the UHV chamber and transmitted through a viewport. Then, light was refocused again outside the UHV chamber by the lens and transmitted through an optical fiber guided to photodetectors. As photodetectors we used either a photon counting system that consists of a photomultiplier tube (Hamamatsu Photonics, R943-02; wavelength detection range, 160–930 nm) or a grating spectrometer (Roper Scientific, SpectraPro-300i) equipped with a liquid-N_2_-cooled charge-coupled device camera (Roper Scientific, Spec-10:100B/LN; detection range, 200–1100 nm). As a substrate, we used commercially available 300-nm-thick Au(111) films evaporated on mica (Georg Albert PVD). The Au(111) substrate was cleaned by repeated cycles of 0.7 keV Ar^+^ ion sputtering for 30 min and annealing at 800 K for 15 min. The cleanliness of the substrate was verified by obtaining the herringbone reconstruction of the Au(111) surface by STM. The (M)-[7]TH-diol molecules were synthesized and enantiomerically purified by the methods described previously [19]. (M)-[7]TH-diol molecules were deposited inside the preparation chamber by sublimation onto the Au(111) or C_60_/Au(111) substrates using an organic deposition evaporator (Kitano Seiki Co., Tokyo, Japan) containing a boron nitride crucible. Prior to the deposition, the compounds were carefully degassed and the deposition of the (M)-[7]TH-diol molecules were carried out onto surfaces precooled to 150 ± 20 K. All STM measurements were carried out at 79 K under pressures of better than 1.0 × 10^−8^ Pa in a constant current mode using an electrochemically etched Pt/Ir tip (Unisoku Co., Hirakata, Japan). Finally, STM images were analyzed using WSxM 5.0 Develop 7.0 software [20]. The piezo-scanner of STM was calibrated by obtaining the atomic resolution images of Au(111) and Ag(111) surfaces.

## 4. Conclusions

We investigated light emission from (M)-[7]TH-diol molecules adsorbed on the Au(111) surface by STM-LE. Despite the high tunneling current and bias voltage set point, individual (M)-[7]TH-diol molecules adsorbed on the Au(111) surface were clearly imaged. Occasionally, we observed the plasmon light emission enhancement from the (M)-[7]TH-diol molecules adsorbed on the Au(111) surface. However, the plasmon light emission from the (M)-[7]TH-diol molecules adsorbed at the edges of the Au(111) terraces was always strongly suppressed. 

We utilized a C_60_ layers adsorbed on Au(111) as a decoupling buffer layer to avoid luminescence quenching of excited molecules and to suppress strong plasmon light emission from the Au(111) surface. We found that the photon integration map shows a very clear contrast between the Au(111) substrate and the C_60_ islands, indicating strong plasmon light emission suppression due to the presence of the C_60_ buffer layer. We found that (M)-[7]TH-diol molecules preferentially adsorb on the Au(111) surface rather than on the C_60_ islands at a low coverage. To adsorb (M)-[7]TH-diol on the C_60_ islands, we deposited a multilayer of (M)-[7]TH-diol molecules. The photon integration map taken on an (M)-[7]TH-diol multilayer deposited on the C_60_/Au(111) surface shows strong light emission. We obtained the STM-LE spectra to gain insight about the origin of light from the multilayer of (M)-[7]TH-diol adsorbed on C_60_ grown on the Au(111) surface. The STM-LE spectra taken on (M)-[7]TH-diol and C_60_ islands adsorbed on Au(111) show strong plasmon light suppression, whereas the STM-LE spectra taken on the multilayer of (M)-[7]TH-diol molecules deposited on the C_60_/Au(111) surface show strong light emission with meaningful fine structures. It is suggested that the light emission from the (M)-[7]TH-diol multilayer has a plasmon origin, but it is strongly affected by electronic structures and their vibronic transitions in (M)-[7]TH-diol molecules.

## Data Availability

Not applicable.
